# Serum caffeine concentrations in preterm infants: a retrospective study

**DOI:** 10.1038/s41598-023-37544-9

**Published:** 2023-06-26

**Authors:** Masashiro Sugino, Toru Kuboi, Yuta Noguchi, Katsufumi Nishioka, Yoko Tadatomo, Nana Kawaguchi, Takaaki Sadamura, Akiko Nakano, Yukihiko Konishi, Kosuke Koyano, Shinji Nakamura, Hitoshi Okada, Susumu Itoh, Takashi Kusaka

**Affiliations:** 1 Division of Neonatology, NHO Shikoku Medical Center for Children and Adults, 2-1-1 Senyu Cho, Zentsuji City, Kagawa 765-8507 Japan; 2 Department of Pediatrics, Faculty of Medicine, Kagawa University, Kitagun, Kagawa Japan; 3 Division of Analytical Technology, Department of Medical Technology, Kagawa Prefectural University of Health Sciences, Takamatsu City, Kagawa Japan

**Keywords:** Medical research, Paediatric research

## Abstract

Therapeutic drug monitoring is generally unnecessary in caffeine treatment for apnea of prematurity, as serum caffeine concentrations in preterm infants are normally markedly lower than those at which caffeine intoxication occurs. However, several studies have reported preterm infants having developed toxicity. This retrospective observational study, conducted at a tertiary center in Kagawa, Japan, aimed to evaluate the correlation between the maintenance dose and serum caffeine concentrations and determine the maintenance dose leading to suggested toxic caffeine levels. We included 24 preterm infants (gestational age, 27 ± 2.9 weeks; body weight, 991 ± 297 g) who were treated with caffeine citrate for apnea of prematurity between 2018 and 2021, and 272 samples were analyzed. Our primary outcome measure was the maintenance dose leading to suggested toxic caffeine levels. We found a positive correlation between caffeine dose and serum caffeine concentrations (*p* < 0.05, *r* = 0.72). At doses of ≥ 8 mg/kg/day, 15% (16/109) of patients had serum caffeine concentrations above the suggested toxic levels. Patients who receive doses ≥ 8 mg/kg/day risk reaching the suggested toxic serum caffeine levels. It remains unclear whether suggested toxic caffeine concentrations are detrimental to neurological prognosis. Further investigation is required to understand the clinical effects/outcomes of high serum levels of caffeine and to obtain long-term neurodevelopmental follow-up data.

## Introduction

Caffeine citrate is a potent therapeutic agent for apnea of prematurity. Caffeine citrate has been reported to reduce mortality from chronic lung diseases and improve neurological prognosis^1^.

Several studies have recently investigated early high-dose caffeine treatment^2–5^ to determine the enhancing effect of such treatment. However, despite reporting improvements in respiratory function, an increased risk of cerebellar hemorrhage post-caffeine treatment and the need for caution with the early use of caffeine have also been noted^4,5^. No consensus has been reached concerning the timing of caffeine treatment, and questions remain regarding the benefits of early caffeine treatment, particularly in terms of facilitating early extubation in mechanically ventilated infants^5^. Most studies have not measured serum caffeine concentrations; therefore, it remains unclear whether the serum caffeine concentrations reported in these studies were below suggested toxic levels.

Therapeutic drug monitoring (TDM) is generally considered unnecessary for caffeine treatment in preterm infants. This is because the suggested toxic caffeine concentration level (50 mg/L) has been reported to be significantly higher than the effective serum caffeine concentration level in preterm infants (5–30 mg/L)^6,7^. It has been reported that serum caffeine concentration levels higher than the standard maintenance dose (5–10 mg/kg/day of caffeine citrate) rarely exceed the effective serum caffeine concentration; however, we previously reported cases in which preterm infants developed suggested toxic serum caffeine concentrations despite receiving a standard dose (10 mg/kg/day)^8^. Therefore, this study aimed to evaluate the correlation between the maintenance dose and serum caffeine concentrations and to determine what dosage level leads to suggested toxic caffeine levels, using a larger sample than in previous studies. Moreover, we aimed to determine whether there would be a difference in serum caffeine concentration levels between extremely preterm infants (age < 28 weeks gestation) and early preterm infants (age ≥ 28 weeks gestation) as, to our knowledge, no clinical unit conducts TDM for caffeine in extremely preterm infants.

## Methods

### Participants

Our study comprised preterm infants aged < 35 weeks gestation who had been treated with caffeine citrate for apnea of prematurity in our neonatal intensive care unit from July 2018 to April 2021. We excluded infants with congenital malformations (e.g., hydrocephalus) or chromosomal abnormalities (e.g., trisomy 21). This study was approved by the Research and Ethics Committees of the Shikoku Medical Center for Children and Adults (No. R02-05). All methods were performed in accordance with the relevant guidelines and regulations. The need for informed consent was waived by the Ethics Committee of the Shikoku Medical Centre for Children and Adults (No. R02-05).

Caffeine citrate was administered at a loading dose of 20 mg/kg/day and a maintenance dose of 5–10 mg/kg/day. The normal caffeine concentration was defined within the therapeutic range (5–30 mg/L), which is considered as the international standard^6,7^. Suggested toxic concentration was defined as a concentration at which serious adverse effects have been reported to have occurred (≥ 50 mg/L)^9–11^. A high concentration was defined as a concentration between the effective and toxic concentrations. As in previous studies, symptoms of caffeine intoxication were defined in relation to gastrointestinal, cardiovascular, and central nervous system symptoms. Gastrointestinal symptoms included nausea and vomiting, and cardiovascular symptoms included tachycardia, which has been defined as a heart rate > 180 beats per minute. Central nervous system symptoms included irritability and tonic posturing^7^.

### Samples

In our neonatal intensive care unit, residual sera collected during standard treatment were cryopreserved at 203.15 K. More specifically, serum remaining after use for routine testing were cryopreserved within 1 h. We used cryopreserved sera collected from the participants 2–3 h prior to caffeine administration. All samples were taken after at least two maintenance doses were administered or after an increase in the maintenance dose. This was to ensure that the maintenance dose initiation or dose escalation was reflected in serum concentrations. Some samples were collected from the same individual. The number of measurements per patient differed owing to the exhaustive use of stored samples. The number of samples per patient was 11.3 ± 6.7. We did not distinguish whether the route of caffeine administration was enteral or parenteral, as the bioavailability of caffeine is almost 100% by enteral administration.

### Measurement

Serum caffeine concentrations were measured using high performance liquid chromatography (HPLC). HPLC consists of a pump (LC20AB, Shimadzu, Kyoto, Japan), an autosampler (SIL20AC, Shimadzu), a column (Kinetex C18 [5 µm 150 × 4.5 mm], Pnenomenex, California, USA), a column oven (CTO20AC, Shimadzu), and a UV/VIS detector (SPD20A, Shimadzu). The measurements were conducted in accordance with Berlin et al.’s study^12^. Isocratic elution was performed using 90% 0.1 M sodium acetate buffer (pH 4.0) and 10% acetonitrile (HPLC grade) with a mobile phase flow rate of 1 mL/min. The measurement wavelength was 280 nm. The internal standard method was used, and 8-chlorotheophuyline was used as the internal standard. The limit of quantification was 0.092 mg/L when the S/N ratio was set to 10. Serum without caffeine intake was used for calibration curve preparation, and caffeine concentrations of 1.5, 3.1, 6.2, 12.5, 25, 50, and 100 mg/L were used for calibration curve preparation. The stored serum was thawed at the time of measurement and, after adding 3 µL of the internal standard, 30 µL of acetonitrile, and 30 µL of distilled water to 30 µL of serum, the mixture was centrifuged at 12,000 rpm for 5 min, and 10 µL of the supernatant was injected into the HPLC.

### Statistical analysis

Statistical sample size calculations were not performed as, in this pilot study, we used a retrospective, exhaustive survey of existing samples. However, the sample size of 109 samples in the < 8 mg/kg group and 163 samples in the > 8 mg/kg group gave a post hoc power of 99% in detecting differences with a mean of 10%, using a two-group Mann–Whitney U test with a two-sided significance level of *p* < 0.05.

Continuous data were tested for normality using a Kolmogorov–Smirnov test and expressed as mean ± standard deviation or median with interquartile range, as appropriate. An F-test was used to confirm that they were not equally distributed. The correlation between serum caffeine concentrations and administered caffeine was tested using Pearson's product-moment correlation coefficient. Doses that resulted in high serum caffeine concentrations and doses that were within the normal concentration were compared using a Mann–Whitney U test. Independent samples from three groups were compared using a Kruskal–Wallis test, a non-parametric test method. A chi-square test was used in the comparison of proportions. All *p* values were two-sided, and *p* values ≤ 0.05 were considered statistically significant. All analyses were performed with EZR version 1.54 (Saitama Medical Center, Jichi Medical University, Saitama, Japan) software, which is a graphical user interface for R (The R Foundation for Statistical Computing, Vienna, Austria).

## Results

**Figure 1:**
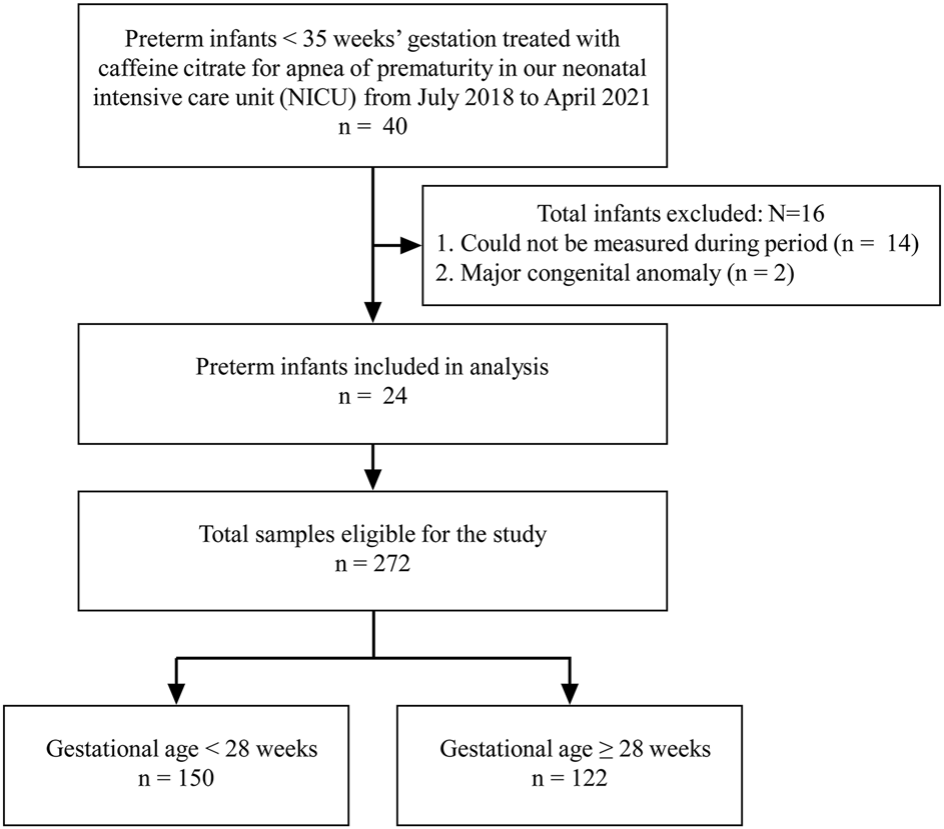
Recruitment and enrollment of study participants.

**Figure 2:**
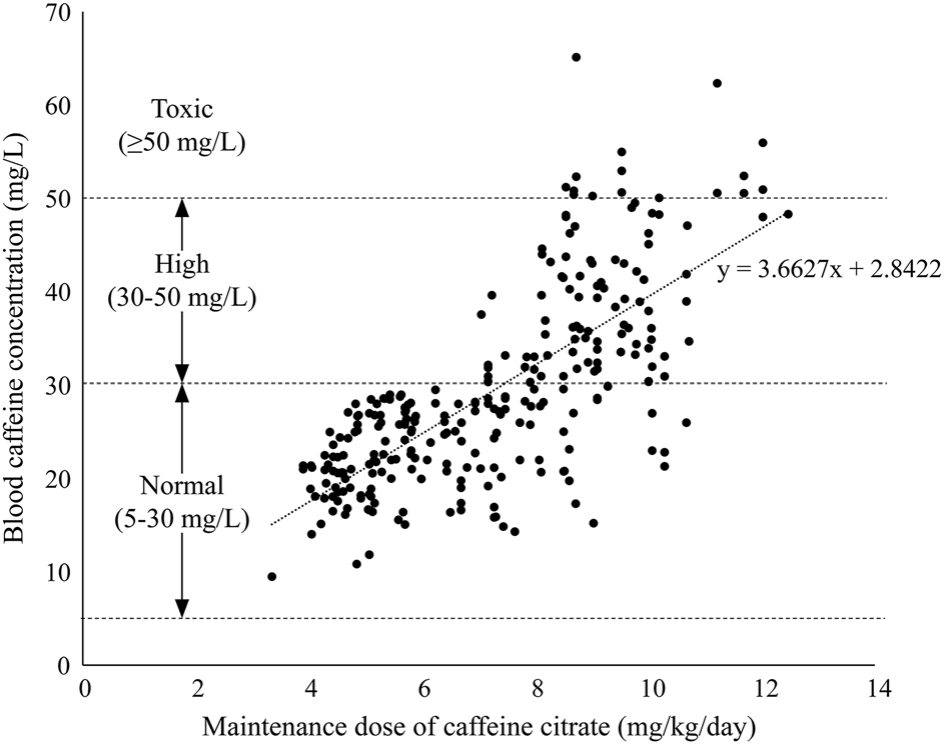
The correlation between dose per body weight and serum caffeine concentrations.

Twenty-four preterm infants (gestational age, 27 ± 2.9 weeks; body weight, 991 ± 297 g) were included in this study, and 272 samples were collected (Fig. 1). Of these, 150 samples were collected from 10 infants aged < 28 weeks gestation and 122 samples from 14 infants aged ≥ 28 weeks gestation. There was a positive correlation between dose per body weight and serum caffeine concentrations (*p* < 0.05, *r* = 0.72) (Fig. 2). At a maintenance dose of < 8 mg/kg, there were no samples of suggested toxic concentrations (0/163). However, at a maintenance dose of ≥ 8 mg, 15% (16/109) of samples exceeded the suggested toxic concentration. Serum caffeine concentrations at doses of ≥ 8 mg/kg were significantly higher than those at doses of < 8 mg/kg (38.2 ± 9.9 mg/L vs. 23.3 ± 5.1 mg/L, *p* < 0.05).

Of 272 samples obtained from 24 patients (27 ± 2.9 weeks of age, 991 ± 297 g), we found a positive correlation between caffeine citrate dose and serum caffeine concentration (*p* < 0.05, *r* = 0.72), with significantly higher serum caffeine concentrations observed at doses ≥ 8 mg/kg than at doses < 8 mg/kg (38.2 ± 9.9 mg/L vs. 23.3 ± 5.1 mg/L, respectively; *p* < 0.05).

Of samples at doses of < 8 mg/kg/day, 93% (151/163) showed a serum caffeine concentration within normal levels (therapeutic range), and none exceeded suggested toxic concentration levels whereas, of samples at doses of ≥ 8 mg/kg/day, only 23% (25/109) were within normal concentration levels, 77% (84/109) were above normal concentration levels, and 15% (16/109) were above suggested toxic concentration levels (Table 1). At a dose of < 8 mg/kg, the samples with high serum caffeine concentration had significantly lower birth weights and significantly lower gestational ages. There was no significant difference in postmenstrual age. At a dose of ≥ 8 mg/kg, there was no significant difference in birth weight or gestational age between the normal and suggested toxic concentration samples. Postmenstrual age was significantly lower in samples with suggested toxic concentrations.

**Table 1:** Characteristics of samples in relation to doses < 8 mg/kg and ≥ 8 mg/kg.

	< 8 mg/kg (n = 163)		*p* value	≥ 8 mg/kg (n = 109)			*p* value
	Normal (n = 151)	High (n = 12)		Normal (n = 25)	High (n = 68)	Toxic (n = 16)	
Birth weight (g)	947 ± 281	738 ± 76	< 0.05	741 ± 197	875 ± 233	801 ± 150	0.23
Gestational age (weeks)	27.2 ± 2.8	24.7 ± 0.3	< 0.05	25.6 ± 2.5	26.5 ± 2.3	26.3 ± 1.9	0.19
Postmenstrual age (weeks)	31.8 ± 2.5	30.6 ± 3.5	0.12	31.0 ± 2.4	31.2 ± 1.6	29.5 ± 1.1	< 0.05
Maintenance dose (mg/kg/day)	5.6 ± 1.0	7.4 ± 0.3	< 0.05	8.9 ± 0.7	9.3 ± 0.8	10.0 ± 1.3	< 0.05
Serum caffeine concentration (mg/L)	22.5 ± 4.4	32.9 ± 2.8	< 0.05	26.1 ± 6.3	39.1 ± 5.6	53.2 ± 4.4	< 0.05

*p value for comparison between normal concentration and toxic concentration.

Ninety-three percent (151/163) of samples at doses of < 8 mg/kg/day within the normal concentration and none exceeded the suggested toxic concentration, whereas 77% (84/109) of patients had doses of ≥ 8 mg/kg/day above the normal concentration, of whom 15% (16/109) were above the suggested toxic concentration. A Kruskal–Wallis test was used for statistical analysis of comparisons among the three groups at doses ≥ 8 mg/kg.

In the analysis based on gestational age, at doses of < 8 mg/kg, 100% (81/81) of the samples with a gestational age ≥ 28 weeks were within the normal range (Table 2). In contrast, 85% (70/82) of the samples with a gestational age < 28 weeks were within the normal range, and this difference was statistically significant (*p* < 0.05). At a dose of ≥ 8 mg/kg, 15% (6/41) of the samples with a gestational age ≥ 28 weeks were within the normal range, 68% (28/41) had high serum caffeine concentration levels, and 17% (7/41) were above suggested toxic concentration levels. In contrast, 28% (19/68) of the samples with a gestational age < 28 weeks were within the normal range, 59% (40/68) had high serum caffeine concentration levels, and 13% (9/68) were above suggested toxic concentration levels; however, the differences were not statistically significant.

**Table 2:** Analysis according to gestational age of the samples in relation to doses < 8 mg/kg and ≥ 8 mg/kg.

	< 8 mg/kg			≥ 8 mg/kg		
	< 28 weeks (n = 82)	≥ 28 weeks (n = 81)	*p* value	< 28 weeks (n = 68)	≥ 28 weeks (n = 41)	*p* value
Normal, % (n/total n)	85 (70/82)	100 (81/81)	< 0.05	28 (19/68)	15 (6/41)	0.15
High, % (n/total n)	15 (12/82)	–	–	59 (40/68)	68 (28/41)	0.41
Toxic, % (n/total n)	–	–	–	13 (9/68)	17 (7/41)	0.58

At a dose of < 8 mg/kg, 100% (81/81) of the samples from preterm infants aged ≥ 28 weeks gestation were within the normal concentration. However, 85% (70/82) of the samples from those aged < 28 weeks gestation were within the normal concentration, and this difference was found to be significant (*p* < 0.05). At a dose of ≥ 8 mg/kg, 15% (6/41) of the samples from ≥ 28 weeks were within the normal concentration range, and 17% (7/41) were above the suggested toxic concentration. However, 28% (19/68) of the samples from < 28 weeks were within the normal concentration, and 13% (9/68) were above the suggested toxic concentration. These differences were not statistically significant. A chi-square test was used in the comparison of proportions.

In all-sample analysis, there was no significant correlation between serum caffeine concentrations and birth weight, gestational age, or postmenstrual age (*r* = − 0.09 and *p* = 0.136; *r* = − 0.11 and *p* = 0.05; *r* = − 0.15 and *p* < 0.05, respectively). The patients with serum caffeine concentrations above the suggested toxic concentration did not show obvious toxic symptoms. No apparent adverse effects (tachycardia, increased reflux episodes, increased feed intolerance, or delay in achieving full feeds) could be attributed to caffeine. Furthermore, no intracranial hemorrhage or periventricular leukomalacia was observed after caffeine administration at the time of discharge. There was only one case of cerebellar hemorrhage. The cause of the cerebellar hemorrhage was unclear but may have been influenced by the infant being born extremely prematurely. The maximum dose in this case was 9 mg/kg, and the maximum serum concentration was 35.7 mg/L, not exceeding the suggested toxic level (50 mg/L). Neurodevelopmental data at the time of discharge were not assessed.

## Discussion

TDM is generally considered unnecessary for caffeine treatment of apnea of prematurity in preterm infants. This is because the caffeine concentration at which toxicity occurs is markedly higher than the effective serum caffeine concentration. While several studies have previously measured serum caffeine concentrations, doses were not evaluated^13,14^. In undertaking dose evaluation, this study showed that an administration of 8 mg/kg/day of caffeine citrate may lead to suggested toxic serum caffeine concentrations. However, some patients receiving this dose had caffeine concentrations within the normal range, which may be due to individual differences in caffeine excretion.

In preterm infants, especially in the neonatal period, urinary metabolic pathways are mainly responsible for caffeine metabolism^15^. In adults, caffeine is metabolized and excreted by hepatic CYP1A2. However, in preterm infants, caffeine is not metabolized because of low CYP1A2 activity. Furthermore, due to reduced reabsorption in the renal tubules, most caffeine is excreted unchanged in the urine^15^. Therefore, the lower the urinary excretion, the higher the serum caffeine concentration. The determinants of urinary excretion are the glomerular filtration rate (GFR), renal tubular secretion, and reabsorption in the renal tubule. In preterm infants, most caffeine is not reabsorbed or secreted in the renal tubules. Caffeine excretion is therefore only affected by the GFR^16^. Therefore, individual differences in serum caffeine concentrations at the same dosage can be considered to reflect differences in the GFR. Future studies should evaluate the correlation between the GFR and serum caffeine concentration levels using serum creatinine and cystatin C, which are used to estimate the GFR. It was expected that the lower the gestational age of the infant, the higher the serum caffeine concentration, as it has been reported that the lower the gestational age, the lower the GFR^12^. However, in this study, there was no significant correlation found between serum caffeine concentrations and gestational age or postmenstrual age. This may have been due to individual differences in the GFR, based on the treatment course, regardless of gestational age or postmenstrual age. At a dose of ≥ 8 mg/kg, the postmenstrual age was significantly lower in the toxic concentration group than in the normal group, but the maintenance dose was also significantly higher. Thus, we consider that a lower postmenstrual age may not be the only risk factor for toxic concentration.

In addition, patients who developed suggested toxic levels did not have significant toxic symptoms (e.g., irritability, tachycardia, and polypnea). All reported neonate patients with caffeine intoxication have comprised those who had a single overdose of caffeine^9–11^. A minimum of 7 days was observed before our study patients reached serum caffeine concentrations that might lead to caffeine intoxication. The lack of obvious caffeine intoxication symptoms may have been because of the absence of a rapid increase in serum caffeine concentrations. Even though the concentrations did not rise rapidly, no obvious symptoms were observed in cases above the suggested intoxication level. Therefore, it may not be appropriate to uniformly describe concentration levels ≥ 50 mg/L as indicative of caffeine intoxication but rather as caffeine levels exceeding the suggested threshold for toxicity.

This study had some limitations. In this pilot study, stored sera were extensively measured. We did not plan the sample size and we reported the results for all the samples included in our study. Future studies should include calculations of sample number and plan accordingly. In addition, since this study was an exhaustive survey, gestational age, the age at the time of caffeine administration, and the duration of caffeine administration varied. Based on our findings, samples at doses ≥ 8 mg/kg/day were likely to exceed the suggested toxic serum caffeine concentrations; therefore, we intend to increase the number of samples at doses ≥ 8 mg/kg/day in a future study.

The effects of high serum caffeine concentrations on neurological prognosis remain to be elucidated. However, several studies have reported that high serum caffeine concentrations are detrimental to brain neurons^17–19^. According to these studies, in terms of mechanisms involved in vitro, caffeine induces apoptotic neuronal death and inhibits cholesterol synthesis by glial cells. Caffeine decreases the number of proliferating glial cells and affects the composition of the extracellular matrix, which could impair myelination. In vivo, postnatal caffeine treatment might induce an alteration of astrocytogenesis via A2aR blockade during brain development. Moreover, early exposure of suckling offspring to caffeine could impair the normal development of the cerebellum through changing the profiles of long-chain saturated fatty acids and long-chain monounsaturated fatty acids. Therefore, our findings suggest that patients with high serum caffeine concentrations require long-term developmental follow-up.

## Conclusion

Our findings indicated that patients receiving maintenance doses of 8 mg/kg/day have an approximately 80% risk of developing serum caffeine levels above normal concentration levels and a 15% risk of developing serum caffeine levels above suggested toxic concentration levels. It remains unclear whether suggested toxic serum concentrations are detrimental to neurological prognosis. Further investigation is required to understand the clinical effects/outcomes of high serum levels of caffeine and to obtain long-term neurodevelopmental follow-up data.

## Data Availability

The datasets used and/or analyzed during the current study are available from the corresponding author on reasonable request.
